# Exploring problems for school reintegration following spinal cord injury: Perspectives on the kindergarten through fifth-grade population

**DOI:** 10.3389/fresc.2022.962682

**Published:** 2022-08-31

**Authors:** Brooke Reeves, Rebecca Martin

**Affiliations:** ^1^ International Center for Spinal Cord Injury, Kennedy Krieger Institute, Baltimore, MD, United States; ^2^ Department of Physical Medicine and Rehabilitation, Johns Hopkins School of Medicine, Baltimore, MD, United States

**Keywords:** education, spinal cord injury, rehabilitation, return to school, pediatric

## Abstract

When a child acquires a spinal cord injury or disorder (SCI/D), they are faced with sudden onset of changes. Engagement in education, play, and leisure is immediately impacted. Using survey methodology, return to school for children in kindergarten through fifth grade following SCI/D was examined. Families at a large spinal cord rehabilitation center for the pediatric population on the East Coast of the United States were surveyed *via* email about their child's return to school to understand support and barriers a child faces when returning to school following SCI/D. Survey findings indicated that children face barriers related to school access, transportation, and educational support upon return to school. The primary barrier identified was access to educational support. Using survey findings, an in-clinic outpatient return-to-school recommendation form was developed to bridge the gap between medical model therapists and school staff that is specific to each child's needs. The curriculum is being developed to support families and therapists in preparing a child to return to school following SCI/D and to educate families to advocate for their child in the school setting. Future recommendations include further research regarding the placement of children in the school environment following SCI/D.

## Introduction

Approximately 294,000 Americans are living with SCI/D, and on average, 17,810 new spinal cord injuries occur each year (1). Children are not excluded from acquiring SCI/D. In fact, approximately 20% of SCI/D occurs in the pediatric population (2). Pediatric SCI/D presents substantial challenges that are different from adult SCI/D as children are still maturing physically, mentally, and emotionally (3, 4). In the United States, the leading cause of pediatric SCI is motor vehicle accidents, followed by falls (5). Other frequently cited causes of pediatric spinal injuries are birth injuries, sports injuries, diving injuries, pedestrian injuries, and gunshot wounds (2, 6).

Individuals with SCI/D often face various issues accessing and engaging in daily occupations, limiting their quality of life. Children with SCI/D often experience a sudden onset of dysfunction in the school environment as a direct result of their injury or illness, resulting in limited participation. Younger children, when compared to adolescents, are more likely to have injuries that result in paraplegia (2). It should be easier to reintegrate a child with paraplegia back into the school environment as they are likely to have fewer medical complications than a child who experiences tetraplegia.

Although education is a priority in a child's life, there is little research regarding pediatric SCI and return to school (7, 8). In fact, the literature that is presented from 1991 to the present day primarily addresses the adolescent population, adults with SCI, or the medical management, rehabilitation, functional levels of independence, etiology, and psychosocial ramifications of a spinal cord injury (9). Not only is the literature scarce but also old; the most relevant sources relating to return to school and SCI are from 1991 to 1999. This is surprising as the prevalence of SCI/D has increased. Additionally, no direct sources are addressing the need to return to school following SCI/D for the kindergarten through fifth-grade population. The reintegration of children into the elementary school setting following SCI/D has not been addressed in the literature.

In a retrospective review of the literature, Sanford et al. (8) examined patients 18 years and younger living with SCI who participated in inpatient rehabilitation from 1989 to 1995 to identify barriers present when returning to the school environment. Sanford et al. (8) found multiple barriers that impeded the ability to return to school; however, architectural barriers were the most frequently identified. As a result, children indirectly have had their independence taken away by barriers they cannot control in the environment. Dudgeon et al. (7) additionally noted barriers impacting children's return to school following SCI/D. Primary barriers identified within this study aligned with those found by Sanford et al. (8). Barriers including architectural barriers, wheelchair accessibility, transportation accessibility/problems, difficulty completing required schoolwork, and stigma and attitudes of school staff or peers have been identified regarding return to school for the adolescent population (7). Overall, as pediatric SCI/D continues to evolve and increase in incidence, it is important that the roles of a child such as play, leisure, social engagement, and education are addressed following initial hospitalization.

## Methods

To identify barriers and supports that exist in returning a child to school following SCI/D, we aimed to explore return to school by surveying our existing patients to improve our service provision and validate the experiences described in the literature. Families with children ages 5–12 years, injured between kindergarten through fifth grade, were surveyed about the child's injury level, classification, mechanism of injury, demographic information, including age at injury and age at the survey, therapy services received, and educational placement in the school environment.

The survey underwent multiple revisions before being distributed electronically to families. Following the initial design of the survey, preliminary drafts were reviewed by a panel of experts to ensure that the survey was clear and understandable for families as well as to ensure the validity of questions. From initial comments, changes were made and presented to the panel for final review prior to being sent to families. Utilization of the panel increased the validity of the survey and helped to ensure what was intended to be studied was being studied. For the purpose of this survey, educational settings were defined using the International Spinal Cord Society (ISCOS) basic data set for education. Regular education was defined as an inclusive setting where resources and support are provided to support functions within the typical classroom (10). Integrated education was defined as separate classes and additional resources for children with SCI within the regular school environment (10).

Participants were recruited at a large spinal cord rehabilitation center for the pediatric population on the East Coast of the United States. Inclusion criteria included parents with children ages 5–18 years, with spinal cord injury or illness at all neurological levels, and who were previously enrolled in kindergarten through fifth grade prior to the injury. Children who did not previously attend school prior to injury or who have a concomitant diagnosis affecting cognition such as a traumatic brain injury were excluded. Survey questions aimed to answer the following questions:

1. Following initial hospitalization for an SCI/SCD, how long until a child returns to the grade school environment?
2. What are the supports and barriers for children with SCI/SCD returning to the school environment following injury?
3. Were families provided adequate information during their child's course of rehab to promote their child's return to the school environment following SCI/D?

The purpose of this paper is the exploration of barriers and facilitators for return to school following SCI/D for the kindergarten through fifth-grade population in preparation for a larger multicenter collaboration.

### Survey findings

Using a documentation database, emails of 135 families with children under the age of 18 years were gathered during the initial phase of participant recruitment. A total of 126 surveys went out; nine families did not receive a survey secondary to invalid email addresses or no email provided. A total of 42 participants responded to the survey *via* Qualtrics. Of the 42 surveys returned, 17 met inclusion and exclusion criteria and were analyzed using descriptive statistics.

The average age of children at survey administration was 11.94 years ,with a range from 6 to 19 years. The average age at the time of injury was 7.53 years, with a range of 5–12 years. The average number of days a child missed school following their SCI/D was 82 days, with a range from 4 to 150 days. Demographic data regarding injury level and mechanism were collected. When examining the level of injury, 0% of the population responded who had children with higher-level cervical injuries (C1–C4) and lower-level lumbar injuries (L1 and below). Thirty-five percent of children who returned to school had thoracic level injuries (T1–T12) and 12% had C5–C8 level injuries. Children primarily had injuries of a nontraumatic etiology (76%), such as acute flaccid myelitis, transverse myelitis, or Guillain–Barre syndrome.

When analyzing return to school for the K-5 population, it was critical to understand where a child was placed prior to SCI/D and where they landed educationally following SCI/D. Eighty-eight percent of survey respondents had children in the regular education setting (Table 1). Prior to the injury, no children were placed in integrated or special education; 12% of the population was previously enrolled in homeschooling (Table 1).

**Table 1:** 

Educational placement (*n *= 17)		
Educational setting	Prior to injury	Following injury
Regular education	88% (*n* = 15)	47% (*n* = 8)
Integrated education	0%	35% (*n* = 6)
Special education	0%	0%
Home school	12% (*n* = 2)	12% (*n* = 2)

Following SCI/D, the educational placement and educational support needs changed for some children. The largest shift was identified in children who previously were placed in the regular education setting (Table 1). Previously, 88% of children were in the regular education setting; however, following SCI/D, 47% remained in regular education and 35% of children were now placed in integrated education upon their return to school. Fifty-three percent of children required an Individualized Education Plan (IEP) upon return to school, 24% required a 504 plan, and only 12% of children did not require support prior to SCI/D in their educational environment.

A child with a SCI/D often requires support to manage their day-to-day participation in education. Parents were asked about the support their children required upon their return to school. The most common supports required were school physical therapy (47%), followed by school occupational therapy (41%), counseling and mental health services (41%), and adaptive physical education (41%). Additionally, 35% of the population required direct education support such as a 1:1 aide, personal assistant, or paraprofessional.

Parents were asked additionally to identify whether their child faced barriers in school access, transportation, and educational support. The primary barrier found was in relation to school access. Thirty-five percent of parents noted their child experienced barriers in school access, such as difficulty navigating and accessing the school building.

## Discussion

Based on these survey findings, placement following SCI/D stood out as a high number of children were placed in integrated education. Integrated education allows a child to receive special education services in the general education setting. Parents surveyed did not have a child who experienced cognitive decline but had children who were removed from general education and placed in integrated education.

There is literature regarding children with physical disabilities returning to school but nothing regarding a child returning to their premorbid school environment following SCI/D (9). Children who experience SCI/D typically cannot be placed in the same category as children with a physical disability such as cerebral palsy or a traumatic brain injury. Children with SCI/D typically do not have cognitive limitations that result in qualification for special education services and an IEP. A child qualifies for special education services in the United States if their disability results in a lack of educational progress requiring special intervention (11). Children with SCI/D tend to fall off the radar of school staff as they do not meet the qualifications necessary for an educational disability and an IEP. A child might have the ability to qualify under the IEP category of other health impairments or orthopedic impairment if they had an unknown premorbid learning disability or delay that was not identified prior to injury, which in turn impacts their educational progress.

Children can be serviced in the school setting under Section 504 of the Rehabilitation Act of 1973, which allows for accommodations or modifications in the school environment to facilitate access to education (12). Children are provided with a 504 plan when they do not qualify for an IEP but require support for participation in education. On occasion, children in some school districts are often placed on an IEP as opposed to a 504 plan to receive services, as some school districts do not provide direct services such as physical or occupational therapy to children with 504 plans. Schools are also provided with funding for students who have IEPs and are not provided funding for students who have 504 plans. This is an ethical conflict as students must be served for counties to receive funding. As schools are required to provide children with educational modifications and accommodations regardless of IEP or 504 plan, the fact that students experience barriers is unacceptable.

There is little reason why a student may experience barriers in their environment or in accessing their education. Thirty-five percent of students in the present sample experienced educational access barriers, which is a call for concern as schools are required to be accessible for children with disabilities as bound by the Individuals with Disabilities Education Act (IDEA) and the Americans with Disabilities Act (ADA) (11). Providing schools additional education on the needs of children with SCI/D may help to decrease the number of students who experience barriers in their educational pursuit.

It was noted when analyzing data that 41% of parents responded “unknown” regarding their child's level of injury. Parents who responded with unknown may have never been provided education regarding their child's level of injury that can assist with understanding impacts on day-to-day participation. Using this statistics, a call to action for advocacy regarding injury education is raised.

### Limitations

The primary limitation identified within survey data collection was in regard to the limited response rate. Limited data collection could be a result of collecting survey responses during a heightened state of the COVID-19 pandemic. Parents were already stretched thin during this critical time and may have bypassed the survey in the interest of time management and providing for their child.

### Recommendations

Future recommendations include further examination of this specific sample regarding educational placement following SCI/D and examining whether the phenomenon of special education and placement following SCI/D impacts other children outside of those serviced in the specified rehabilitation center. In addition, development of advocacy tools for both parents and therapists will occur in order to promote the success of children with SCI/D in the school environment. Parents require advocacy skills to understand the rights and services their child is entitled to, and therapists require advocacy skills in order to support the child in having appropriate recommendations and accommodations upon return to school.

## Data Availability

The original contributions presented in the study are included in the article/Supplementary Material, further inquiries can be directed to BR reevesb@kennedykrieger.org.
